# The role of the femoral component orientation on dislocations in THA: a systematic review

**DOI:** 10.1007/s00402-021-03982-1

**Published:** 2021-06-08

**Authors:** Joost H. J. van Erp, Thom E. Snijders, Harrie Weinans, René M. Castelein, Tom P. C. Schlösser, Arthur de Gast

**Affiliations:** 1 Department of Orthopedics, Diakonessenhuis, Utrecht, Zeist, The Netherlands; 2 Clinical Orthopedic Research Center m-N, Zeist, The Netherlands; 3 Department of Orthopedics, UMC Utrecht, Utrecht, The Netherlands; 4 Department of Biomechanical Engineering, Delft University of Technology, Delft, The Netherlands

**Keywords:** Total Hip arthroplasty, THA, Femoral component, Orientation, Anteversion, Sagittal tilt, Dislocation, Instability

## Abstract

**Introduction:**

Dislocation remains a major complication in total hip arthroplasty (THA), in which femoral component orientation is considered a key parameter. New imaging modalities and definitions on femoral component orientation have been introduced, describing orientation in different planes. This study aims to systematically review the relevance of the different orientation parameters on implant stability.

**Methods:**

A systematic review was performed according to the PRISMA guidelines to identify articles in the PubMed and EMBASE databases that study the relation between any femoral component orientation parameters and implant stability in primary THA.

**Results:**

After screening for inclusion and exclusion criteria and quality assessment, nine articles were included. Definitions to describe the femoral component orientation and methodologies to assess its relevance for implant stability differed greatly, with lack of consensus. Seven retrospective case–control studies reported on the relevance of the transversal plane orientation: Low femoral- or low combined femoral and acetabular anteversion was statistical significantly related with more posterior dislocations, and high femoral- or combined femoral and acetabular anteversion with anterior dislocations in two studies. There were insufficient data on sagittal and coronal component orientation in relation to implant stability.

**Conclusion:**

Because of incomparable definitions, limited quality and heterogeneity in methodology of the included studies, there is only weak evidence that the degree of transverse component version is related with implant stability in primary THA. Recommendations about the optimal orientation of the femoral component in all three anatomical planes cannot be provided. Future studies should uniformly define the three-dimensional orientation of the femoral component and systematically describe implant stability.

## Introduction

Total hip arthroplasty (THA) is one of the most frequent orthopedic procedures and has been declared the most successful operation of the twentieth century [1], being very effective in relieving pain and improving hip function. Since the introduction of THA, dislocation has been a common complication. After decades of technological improvements in orthopedic surgery, this remains a serious problem, with long-term consequences for the patient. The risk of dislocation after primary THA for osteoarthritis (OA) has been reported 0.3–10% [2]. After a first dislocation, 60% of the patients sustain recurrent instability and 50% of these need revision surgery [3]. Every year almost 50.000 THAs are revised in the United States because of instability, the most common indication for revision (17.3%) [4, 5]. A dislocated THA results in a tripling of the hospital costs, compared to an uncomplicated THA [6].

In the coronal plane, a neutral orientation of a straight stem is traditionally aspired, since a varus/valgus alignment is associated with poorer clinical outcomes, but recent studies do not confirm this [7, 7, 7]. Reconstruction of the femoral offset is another frequently studied parameter. Optimal reconstruction of the anatomical offset is related to reduction of wear and improvement of range of motion [10].

Traditionally, a “safe zone” between 10° and 15° anteversion of the neck of the femoral stem in the transverse plane was used as a guideline for placement of the femoral component. This zone was stated in 1999 by Tönnis et al. and based on the mean native femoral anteversion (FA) of a group of healthy Germans, but there was a wide range in FA between individuals [11]. Since then, this “safe zone” for FA has been widely accepted and used in daily practice and literature, despite individual differences. In addition to this, Ranawat et al. introduced the concept of combined anteversion (CA), which is the sum of the anteversion of the cup and the stem and is 25°–35° in the native situation for men [12]. For women this was adjusted to 20°–45°, since they naturally have a higher FA, and thus higher CA, which was not taken into account by Tönnis et al.. This resulted in an advised “safe zone” for CA of 25°–50° [13].

New definitions were recently proposed to better describe the alignment in the sagittal plane, such as femoral tilt and anterior offset [14, 14, 14]. They showed that anatomical reconstruction in the sagittal plane is crucial for THA stability [14, 14].

In contrast to the femoral component, the role of the orientation of the acetabular component causing dislocation has been extensively investigated. Recent advances have demonstrated the importance of sagittal spinopelvic-femoral dynamics and the effect of functional pelvic tilt on the functional acetabular cup-to-femoral-component orientation [17, 17]. Studies on the 3-D acetabular cup orientation showed that a majority of the dislocations have an acetabular cup position which resides within the “safe zone” [19]. Recent literature has questioned the validity of the so-called “safe zone” to explain dislocations and the variety of definitions used [20, 20, 22]. This has led to abolishment of the “safe zone”. These insights also request reevaluation of the traditionally advised orientation of the femoral component.

This study aims to systematically review the relevance of the femoral component orientation parameters for implant stability in primary THA. A better insight into the femoral components’ orientation in 3-D could result in a more anatomical placement of it and a substantial decrease in dislocations. For practical purposes, the different femoral component orientation parameters found in the existing literature will be stratified according to the three anatomical planes.

## Material and methods

### Identification and screening

The PRISMA statement for systemic reviews and meta-analyses was used. In June 2019, we performed a systematic search in the PubMed and Embase databases. The search syntax composed of “total hip arthroplasty”,”femoral component”, “dislocation” and “orientation”, and synonyms in the singular and plural, obligatory in the title/abstract. Duplicates were removed. Title and abstract of the publications were screened for the inclusion and exclusion criteria by the first two authors using Rayyan®, Qatar Computing Research Institute, Doha, Qatar. Inclusion criteria were the following: Total hip arthroplasty, any parameter describing the orientation of the femoral component, dislocation rate, written in the English language. Exclusion criteria were as follows: cross-sectional studies, animal studies, biomechanical laboratory studies and computer simulation studies. In case of conflicting judgments between the first and second authors, agreement was achieved by discussion. References of all included full-text articles were checked for additional relevant studies.

### Quality assessment

Since no RCTs were identified in the search, the Newcastle–Ottawa Quality Assessment Scale for cohort and case–control studies was used for quality assessment [23]. Studies with any of the following characteristics were scored as poor quality because of high risk for bias and excluded from further analysis: representativeness of the case was defined as poor when more than 20% of the subjects had revision THA or underwent THA for other indications than primary OA. Adequate follow-up for the question of this study was defined as a minimum of 3 months for > 90% of the subjects. Studies not reporting on the exact method how orientation parameters were measured were defined as an ‘insecure record of ascertainment of exposure’.

Studies with poor quality according to the Newcastle–Ottawa quality assessment were excluded from further analysis, whereas studies with fair or good quality were included.

### Data extraction

From the included studies, the following parameters were extracted: Author, year of publication, journal, study design, number of patients and mean and range of follow-up. The percentage of primary THA and OA was analysed, as well as use of cement and implants, dislocation rate, odds ratio for dislocation and imaging modality used for assessment of component positioning.

Description of the used definitions to determine the component orientation and the potential differentiation between anterior and posterior dislocations were noted and included in this review.

Orientation parameters were structured according to the three orthogonal, anatomical planes.

### Coronal plane parameters

- Mean femoral (medial) offset of the control group versus the dislocation group.
- Mean varus/valgus configuration of the femoral component of the control group versus the dislocation group.

### Transverse plane parameters

- Mean femoral component anteversion of the control group versus the dislocation group.
- Mean combined anteversion of the control group versus the dislocation group.

### Sagittal plane parameters

- Mean sagittal tilt or of the control group versus the dislocation group.
- Mean femoral anterior offset of the control group versus the dislocation group.

## Results

**Fig. 1:**
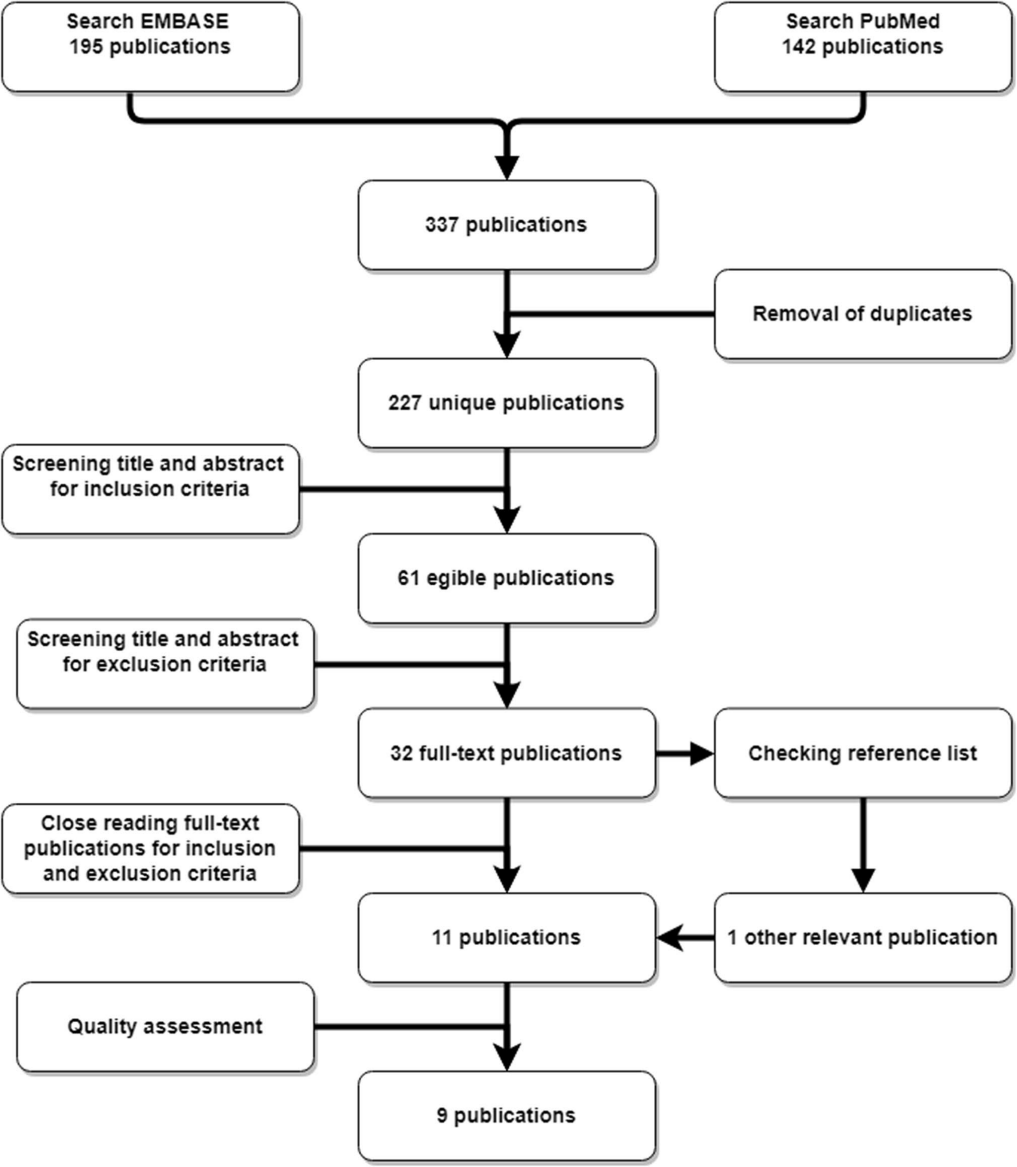
Process of inclusion

The search resulted in 337 publications. After removal of duplicates, 227 remained. By title and abstract screening, 61 articles were selected for full-text screening. Ultimately, nine out of eleven articles met the inclusion and exclusion criteria and met the minimum quality assessment (Fig. 1). Quality assessment according to the Newcastle–Ottawa Quality Assessment scale is displayed in Table 1. Two studies were classified as “fair’’, while the quality of the other seven studies was considered “good”. Two studies (Kawarai et al. and Vrelisovic et al.) were classified as ‘’poor’’ and were, therefore, excluded. The characteristics of the included studies are displayed in Tables 2, 3 and 4. All nine articles were retrospective case–control studies. There were no randomized controlled trials. Regarding publication bias, no overlap was found between authors or institutions. In all studies a straight femoral stem was used.

**Table 1:** Quality assessment according to the Newcastle-Ottawa Quality Assessment scale

Author	Year of publication	Case Definition Adequate	Representativeness Cases	Controls Selection	Controls Definition	Design and Analysis	Outcome Assessment	Equal Ascertainment Cases and Controls	Non-Response Rate	Total Stars/Total Possible Stars	Quality
Dudda et al	2010	+	+	−	+	++	+	+	−	7/9	Good
Fujishiro et al	2016	+	+	−	−	+	+	+	−	5/9	Fair
Jolles et al	2002	+	+	−	+	++	+	+	−	7/9	Good
Kawarai et al	2017	+	-	−	?	+	-	+	−	3/9	Poor
Komeno et al	2006	+	+	−	+	++	+	+	−	7/9	Good
Min et al	2008	+	+	−	+	++	+	+	−	7/9	Good
Nishii et al	2004	+	+	−	+	++	+	+	−	7/9	Good
Pierchon et al	1994	+	+	−	−	+	+	+	−	5/9	Fair
Reina et al	2017	+	+	−	+	++	+	+	−	7/9	Good
Vrelisovic et al	1994	+	-	?	?	+	?	?	?	2/9	Poor
Yoshitani et al	2018	+	+	−	+	++	+	+	−	7/9	Good

The two studies with ‘poor’ quality were excluded for further analyses

**Table 2:** Coronal plane: Characteristics of included studies, RMCC=retrospective matched case control, PL=posterior, AL=anterolateral, MFO=medial femoral offset

Author	Year	Approach	# patients/hips	Radiograph	CT	Mean FU	Design	Disclosures	Dislocation %	Cemented femur	Different components	Sig dif Varus/ valgus	Sig dif MFO
Jolles et al	2002	PL	42	+	−	?	RMCC	No	30/2023 1,5%	?	+		−
Min et al	2008	AL	98 hips	+	−	8 years	RMCC	No	1/98 1%	−	−	−	
Nishii et al	2004	PL	156/191	−	+	>2 years	RMCC	N/A	10/191 5,2%	−	−		−

**Table 3:** Transversal plane: Characteristics of included studies, RCC=retrospective case control, RMCC =retrospective matched case control, PL=posterolateral, AL=anterolateral, AN=anterior, SL=straight lateral, FA=femoral anteversion, CA=combined anteversion

Author	Year	Approach	# patients/hips	Radiograph	CT	Mean FU	Design	Disclosures	Dislocation %	Cemented femur	Different components	Sig dif FA	Sig dif CA
Dudda et al	2010	PL+AL+SL	826	+	−	37 days	RMCC	?	?	?	+	−	−
Fujishiro et al	2016	PL	1555	−	+	53 months	RCC	?	50/1555 3,2%	?	+	+	+
Jolles et al	2002	PL	42	+	−	?	RMCC	−	30/2023 1,5%	?	+	−	−
Komeno et al	2006	PL	38	−	+	>3 years	RMCC	?	?	?	?	−	+
Nishii et al	2004	PL	156/ 191	−	+	> 2 years	RMCC	?	10/191 5,2%	−	−	−	−
Pierchon et al	1994	PL	52	−	+	?	RCC	−	?	−	+	−	−
Reina et al	2017	AN+PL	56	−	+	?	RMCC	+	?	±	+	−	−

**Table 4:** Sagittal plane: Characteristics of included studies, RMCC=retrospective matched case control, PL=posterolateral, AL=anterolateral, AFO=anterior femoral offset

Author	Year	Approach	# Patients/hips	Radiograph	CT	Mean FU	Design	Disclosures	Dislocation %	Cemented femur	Different components	Sig dif AFO	Sig dif sag flexion
Yoshitani et al	2018	AL+PL	89/ 102	+	+	5 years	RMCC	–	3/370 0,8%	–	–	–	–

There were three studies which reported about the femoral component orientation in the coronal plane, seven studies about the transversal plane and two studies about the sagittal plane. In all studies dislocation was the primary outcome. There were no studies in which a power analysis was conducted.

### Coronal plane parameters

#### Varus/valgus

Min et al. (*n* = 98, 2008) reported on the role of varus/valgus alignment of the femoral component on dislocations. They used postoperative upright anterior–posterior (AP) radiographs. Varus/valgus was defined as the angle between the longitudinal axis of the proximal femur and the longitudinal axis of the stem of the femoral component in the coronal plane. When this angle was < 5°, the component was considered in neutral orientation, values > 5° were considered varus/valgus. Min et al. [24] found no statistical significant difference regarding instability, yet reported one dislocation, which was in a varus alignment THA. Valgus was not associated with a higher chance of dislocation.

#### Medial femoral offset (MFO)

There were two studies included that investigated the relation between MFO and dislocations, namely Jolles et al. (*n* = 42, 2002) and Nishii et al. (*n* = 191, 2004). This was defined as the distance from the center of rotation of the femoral head to the central axis of the femur in the frontal plane. No statistical significant increase of instability was seen when MFO was enlarged or reduced, compared to the native situation. Besides, no difference was found when comparing the absolute MFO between stable and unstable hips [25, 25].

### Transversal plane parameters

#### Femoral Anteversion (FA)

Seven articles described the relation between femoral anteversion (FA) and instability, viz. Pierchon et al. (*n* = 52, 1994), Jolles et al. (*n* = 30, 2002), Nishii et al. (*n* = 191, 2004), Komeno et al. (*n* = 38, 2006), Dudda et al. (*n* = 826, 2010), Fujishiro et al. (*n* = 1555, 2016) and Reina et al. (*n* = 56, 2017). Dudda et al. provided no further information about definitions or methods. Jolles et al. used radiographs to measure FA, this was defined as the angle formed by the bottom line of the lateral radiographic plate representing the posterior plane of the condyles and the axis of the prosthetic neck [25]. All other studies performed measurements using CT scans, using the posterior femoral condyles plane as reference. FA was defined as the angle between the axis of the neck of femoral component and the posterior femoral condylar plane.

In a study of Pierchon et al. the mean FA was 16.5° (range − 30–37), in a mixed population of anterior and posterior dislocated THA versus 14° in the control group, which was not statistically significant [27]. Jolles et al. described a FA of 17.2° (range 14.3–20.1), in mostly posterior, dislocated hips, compared to 14.8° (range 11.6–18) in the control group, which differed not statistically significant [25]. Nishii et al. found an not significantly different FA of 29.6° ± 10.3 in posterior dislocated hips, compared with 31.8° ± 17.2 in the control group [26]. Komeno et al. investigated anterior and posterior dislocations, which had an mean CA of 72.2° respectively 27.4°, both statistical significantly different from the control group of 47.8° [28]. Dudda et al. described an optimal FA between 10° and 15°, based on Odd`s ratios for dislocation without mentioning the mean FA.

Fujishiro et al. found a statistically significant lower FA of 33.2° ± 15 in posterior dislocations compared with an FA of 40.0° ± 11.5 in the control group. The anterior dislocated THA had higher FA, which was not statistically significant. A FA < 20° appeared to be a risk factor for dislocation [29]. Finally, Reina et al. found an FA of 14.2° ± 9.9 (range − 19–40) in the unstable group, with an equal distribution of anterior and posterior dislocated hips, which was similar to that in the stable group, 13.4° ± 4.4, (range 0–25) [30].

#### Combined anteversion (CA)

There were six articles that described the relation between the combined anteversion (CA) and instability, viz. Pierchon et al. (*n* = 52, 1994), Jolles et al. (*n* = 42, 2002), Nishii et al. (*n* = 191, 2004), Komeno et al. (*n* = 38, 2006), Fujishiro et al. (*n* = 1555, 2016) and Reina et al. (*n* = 56, 2017). In all articles, CA was defined as the sum of the anteversion of the acetabular component and the FA. Measurements were performed with CT scans, except for Jolles et al. who used radiographs.

Pierchon measured a mean CA of 41° in a group of unstable hips in a population of anterior and posterior dislocated THA, compared with 36.6° in the control group, which was not a statistically significant difference [27]. Jolles et al. found an equal mean CA of 44,8° in unstable, predominantly posteriorly dislocated THA, compared to 44.7° in the control group. However, an CA < 40° or > 60°, appeared highly predictive for dislocations (odds ratio 6.9; 95% confidence interval 1.17–10.9) [25]. Nishii et al. found an mean CA of 49.9° in posterior dislocated hips, compared to 58.7° in the control group, which was not a statistically significant difference [26]. Komeno et al. investigated anterior and posterior dislocations, were an CA of 72.2° respectively 27.4°, both statistical significantly different from the control group of 47.8° [28]. Fujishiro et al. anterior and posterior dislocations, and found an CA of 79.6° ± 18.2 and 53.3° ± 22.4 respectively, both were statistically significant different from the control group of 64.6° ± 15.7. After univariate analysis, a CA of < 40° or > 60° was defined as a risk factor for dislocation [29]. Last, Reina et al. investigated the CA, 33.5° ± 15.7, (range 20–50) in a unstable group with an equal distribution of anterior and posterior dislocated hips, similar to the control group, 35.7° ± 5.1(range -11–60). Their odds ratio calculated for the CA 40°–60° was 0.402; 95% confidence interval 1.17–10.9 [30].

### Sagittal plane

#### Sagittal tilt

There was one study included that investigated the relation between sagittal tilt and dislocations, namely Yoshitani et al. (*n* = 102, 2018). In this study the proximal femoral bone axis was defined as the line between the center of the canal at the lesser trochanter and the center of the canal at the femoral isthmus. Sagittal tilt was measured as the angle between the axis of the prosthesis and the proximal femoral bone axis on the sagittal plane on CT scans. Flexion ≥ 3° was considered as sagittal tilt. Patients were divided in groups, based on the degree of sagittal tilt, ≤ 3°, 4° or 5°. There was no difference in number of dislocations between the different groups of sagittal tilt [31].

#### Anterior femoral offset (AFO)

AFO is a recently introduced definition by Hirata et al. and is defined as the distance from the posterior femoral condylar plane to the center of the femoral head in the sagittal plane. Yoshitani et al. found that a higher sagittal femoral component tilt resulted in a higher AFO; however, this did not result in a higher number of dislocations. There were no studies found which directly investigated the relation between anterior femoral offset and dislocations.

## Discussion

This study is the first systematic review which tried to find evidence for an optimal 3-D orientation of the femoral component in order to avoid THA dislocations. Theoretically, the femoral component can be implanted with six degrees of freedom in the three anatomical planes: the coronal, transverse and sagittal plane. However, in uncemented THA, the surgeon is less able to influence the positioning of the femoral component in three dimensions since this is partially guided by the stem design and the patients femoral anatomy. Based on this systematic review, we can conclude that there is little evidence for the optimal orientation in the transverse plane. In addition, we can conclude that high-quality data on the orientation and position in all three planes, is lacking. A power analysis was missing in all studies, which causes a high likelihood of under powering. Furthermore, the conjoint analysis of anterior and posterior dislocations severely subverts the reliability of the majority of the studies.

The study of Min et al. showed that deviations of the femoral component in the coronal plane did not coincide with dislocation of the hip; however, they had only one dislocation in 98 THAs. In this case the femoral component was implanted in more than 5° varus. Further research is needed to confirm if a femoral component in varus forms a higher risk for dislocation. A neutral orientation of the femoral component in the coronal plane is advised. For the medial femoral offset, no evidence was found for a relation between different values of MFO and instability. This is comparable to the conclusion of DaFine et al. who performed a systematic review on the role of restoration of the MFO and instability[10]; however, recently in three computer studies relations were described between MFO restoration and instability [32, 32, 32]. Since the amount of femoral rotation dictates the MFO measured on a AP radiograph, it cannot be not reliably measured that way. A combination of standardized upright AP and lateral radiographs could solve this problem, but there was no study that used this method.

In the transverse plane, no subgroup analysis was performed in 5/7 studies between anterior and posterior dislocations. FA and CA values of both anterior or posterior dislocations were bundled in the same mean, which impedes the interpretation of the results. Therefore, FA and CA of unstable and stable THA ends with similar values of anteversion. Komeno et al. and Fujishiro et al. were the only two who performed separate analyses and both found significant differences between groups. Low FA was related with a higher chance of a posterior dislocation, and patients with a high FA were at risk for an anterior dislocation. Only Fujishiro et al. were able to demonstrate a significant lower FA in a posterior dislocated group (*p* = 0.0009).

When comparing the results on FA to the generally accepted “safe zone” of 10°–15°, only the studies of Reina et al. and Jolles et al. found a mean FA of stable THA in this range [30]. Other studies reported substantial higher values of anteversion. In a the study of Reina et al., the mean FA was between 10° and 15° in stable and unstable hips. There were no studies that demonstrated FA in the “safe zone” for stable THA and outside the “safe zone” for unstable THA. Based on this systematic review, no advice can be given regarding the optimal femoral component anteversion, there is no evidence for the “safe zone” of 10°–15°.

Only Fujishiro et al. and Komeno et al. performed a subgroup analysis of the CA between anterior and posterior dislocations. Both found a significant higher and lower, respectively, CA compared to the control group. The other studies, without subgroup analysis, did not report differences in anteversion. When regarding the “safe zone” for CA, of 25°–50°, a widespread variation in results was seen. The majority of the studies had a CA in this range, for both stable and unstable hips. In three studies, the control group had a CA > 50°, while the posterior dislocated THA was within the “safe zone”. It could be concluded that THA with a low, or high CA, are prone to dislocations, but the range for this is completely unsure. The recommendations and “safe zones”, used up to now, fail to predict and clarify the majority of dislocations. The recently described femur first technique might improve the orientation of the components, as it adjusts the cup orientation to comply to the femoral component, in the functional pelvic plane [35].

In the sagittal plane, one study described the effect of sagittal flexion. Besides the relatively small group of THA in flexion (*n* = 44), no direct comparison was made between dislocated hips and stable hip. The sagittal alignment of the femoral component might still explain a significant number of dislocations. Muller et al. showed that a change in sagittal femoral component orientation of 5° can result in a change of 10° in functional transverse femoral component orientation [14]. In addition, Hirata et al. described that de AFO and MFO influence the ROM as well. Maximum range of motion was obtained with a AFO between 15 and 25 mm and with a MFO above 32.1 mm [16]. To avoid mechanical impingement, optimal positioning of the components could be calculated, to assure maximum range of motion. Widmer et al. stated that the combined anteversion was optimal when the sum of the cup anteversion and 0.7 times the FA was 37°[36]. D’Lima et al. performed comparable research and stated as well that a stable THA only could be obtained by a certain combinations of CA and cup abduction [37]. However, all three studies mentioned above were computed simulations.

There are some limitations of this systematic review. Patient selection differed largely between the included articles. All patients were retrospectively selected and there was a large heterogeneity between patients regarding age, comorbidity and indication for THA, all confounders for dislocation according to the literature [39]. The preferred surgical approach of the studies included, was not taken into consideration. Although most patients in this review were operated on via a posterolateral approach, use of the anterior or anterolateral approach influences the orientation of the components [40], which can influence the dislocation rate too. This influences the outcomes of this review, since the dislocation mechanism is different in the direct anterior and anterolateral approach and that anterior dislocations occur more frequent, instead of posterior dislocations, compared to the posterolateral approach [41]. The mechanism behind this might be a caused by a combination of factors. Firstly, mean component orientation seems different between different approaches [42, 42, 44]. Secondly, different approaches have different impact on the soft-tissue tensioning and reconstruction [45].

Surgical approach and soft-tissue status are considered major contributing factors to implant stability in THA. Miller et al. and Ponzio et al. both found a higher risk of dislocation in the posterolateral approach compared to the anterior approach, nowadays the two most used techniques [43, 46]. Soft tissue repair, especially tendon-to-bone, results in a decrease of dislocations and a higher Harris Hip score, according to Zhang et al. Moon et al. and Wu et al. [47–49] Use of tissue repair was frequently not described, in combination with different surgical approaches this results in a greater heterogeneity of the included studies.

Reina et al. studied THA with three different approaches, but different approaches were not subanalyzed [30]. Dudda et al. described a dislocation rate which was six times higher when using the posterolateral approach, but did not describe differences in orientation between the approaches [50]. This could possibly be explored with a description of the components alignment in the orthopedic implant registers, combined with a national registration of dislocations. Another influencing factor is the use of cement which was not described in the majority of the included articles.

Two studies from Asia reported an extremely high FA and CA in their stable population [28], Fujishiro et al. 2016). Studies were performed worldwide, but native femoral and CA differ between different races and individuals [51, 51, 51]. Recently, Lazennec et al. described a wide variation of FA in the standing position. Before surgery, more than 80% of the patients did not reside in the “safe zone”; after THA this was raised until 85% [54]. In other research regarding the native FA, a similar variation was found (-15–30°)[55]. Jolles et al. was the only study that measured the anteversion in the upright position, but used an inaccurate measurement method, based on only an AP radiograph. The orientation of the acetabular cup and the femoral component are united in the term CA, but these combinations are not frequently described for the sagittal and coronal plane; however, some recent studies introduced tools to measure this. Komeno et al. showed that a proper orientation of both is necessary to prevent dislocations [28]. Analysis of “combined valgus/varus” or “combined sagittal tilt” might be necessary to fully understand the etiology of instability [56, 56, 56, 56].

Lack of uniformity in describing the femoral component orientation and/or position request causes inability to compare studies. We performed a systematic best-evidence-synthesis, which is considered a good alternative to a meta-analysis for qualitative analyses of very heterogenic studies [60]. Since the mechanism of dislocation is probably completely different for anterior and posterior dislocations, and only two studies reported on this characteristic, we think it would be a significant methodological error to perform a meta-analysis on the studies included in this systematic review. We do think that it is important to describe our findings to the orthopedic world. Better understanding of the 3D-anatomy of the proximal femur and taking into account its geometric variability between patients, as well as spinopelvic dynamics, might very well further improve the results of THA.

Clear definitions in three planes have to be defined. In our opinion, the orientation of the femoral component should be assessed based on the six degrees of freedom in the three anatomical planes and should be applicable to different imaging modalities and patients in different positions. Besides the 3D orientation of the THA components, patient-specific anatomy might provoke instability. Functional alternations, such as spinopelvic dynamics (limited or abnormal pelvic tilt during positional changes) or soft-tissue/bony impingement, are factors which may not be accounted for by changing the components’ orientation solely and should be analysed in case of THA instability, or ideally during preoperative assessment. Since pelvic tilt and femoral rotation significantly influence the 3-D orientation of prosthesis, differences in THA orientation and position could be expected when changing from a standing to a sitting position. Recently, the relation between preoperative and postoperative sagittal spinopelvic femoral alignment and component orientation in THA has been extensively studied [61]. From these studies it was hypothesized that patients with certain pelvic dynamics are at higher risk for THA instability. In this, Buckland et al. showed that patients with previous spinal fusion are at higher risk for THA dislocation (OR 2,93) [62]. Furthermore, Heckmann et al. showed that patients may develop instability despite optimal component orientation because they have abnormal spinopelvic dynamics characterized by restricted pelvic tilt from standing to sitting position [18]. Preoperative analysis of spinopelvic dynamics may decrease the dislocation rate after THA [63]. The advised “safe zone” of 15° FA should be abolished, since THA instability seems to be related to the 3-D orientation of the complete prosthesis, in relation to surrounding structures, e.g. the pelvis or femur and the preoperative situation.

## Conclusions

It could be concluded that a low femoral- or combined anteversion is related with greater risk of a posterior dislocation and that a high femoral- or combined anteversion increases the risk for an anterior dislocation. Because of incomparable definitions, limited quality and heterogeneity in methodology of the included studies, no further advice could be given about the optimal orientation of the femoral component in all three planes. Further agreement on terminology and their precise univocal definition, and research on the role of the 3-D femoral component orientation on dislocations, is mandatory to provide high-quality evidence allowing for recommendations on femoral component position in THA.

## Appendix 1

### Search Terms.

#### Search PubMed: 142 results

(((((((((((femoral stem[Title/Abstract]) OR femoral stems[Title/Abstract]) OR femoral component[Title/Abstract]) OR femoral components[Title/Abstract]) OR stem[Title/Abstract]) OR stems[Title/Abstract]) OR femoral implant[Title/Abstract]) OR femoral implants[Title/Abstract])) AND (((("positioning") OR "orientation") OR "placement") OR "alignment")) AND ((((((total hip arthroplasty[Title/Abstract]) OR total hip arthroplasties[Title/Abstract]) OR total hip replacement[Title/Abstract]) OR total hip replacements[Title/Abstract]) OR total hip prosthesis[Title/Abstract]) OR total hip prostheses[Title/Abstract])))) AND (((dislocation) OR instability) OR luxation).

#### Search Embase: 195 results

('femoral stem':ab,ti OR 'femoral stems':ab,ti OR 'femoral component':ab,ti OR 'femoral components':ab,ti OR 'stem':ab,ti OR 'stems':ab,ti OR 'femoral implant':ab,ti OR 'femoral implants':ab,ti) AND (positioning OR orientation OR placement OR alignment) AND ('total hip arthroplasty':ab,ti OR 'total hip arthroplasties':ab,ti OR 'total hip replacement':ab,ti OR 'total hip replacements':ab,ti OR 'total hip prosthesis':ab,ti OR 'total hip prostheses':ab,ti) AND (dislocation OR instability OR luxation)
